# Integration of data across toxicity endpoints for improved safety assessment of chemicals: the example of carcinogenicity assessment

**DOI:** 10.1007/s00204-021-03035-x

**Published:** 2021-04-08

**Authors:** Federica Madia, Gelsomina Pillo, Andrew Worth, Raffaella Corvi, Pilar Prieto

**Affiliations:** European Commission, Joint Research Centre (JRC), Via E. Fermi 2749, 21027 Ispra, VA Italy

**Keywords:** Chemical safety assessment, Systemic toxicity endpoint, Key characteristic, New approach methodology

## Abstract

In view of the need to enhance the assessment of consumer products called for in the EU Chemicals Strategy for Sustainability, we developed a methodology for evaluating hazard by combining information across different systemic toxicity endpoints and integrating the information with new approach methodologies. This integrates mechanistic information with a view to avoiding redundant in vivo studies, minimising reliance on apical endpoint tests and ultimately devising efficient testing strategies. Here, we present the application of our methodology to carcinogenicity assessment, mapping the available information from toxicity test methods across endpoints to the key characteristics of carcinogens. Test methods are deconstructed to allow the information they provide to be organised in a systematic way, enabling the description of the toxicity mechanisms leading to the adverse outcome. This integrated approach provides a flexible and resource-efficient means of fully exploiting test methods for which test guidelines are available to fulfil regulatory requirements for systemic toxicity assessment as well as identifying where new methods can be integrated.

**Supplementary Information:**

The online version contains supplementary material available at 10.1007/s00204-021-03035-x.

## Introduction

In October 2020, the European Commission adopted its Chemicals Strategy for Sustainability (European Commission 2020), with the main goal to boost innovation for safe and sustainable chemicals, and increase protection of human health and the environment against hazardous chemicals. This is a key opportunity to become a sustainable climate neutral and circular economy by 2050, as stated in the European Green Deal (European Commission 2019).

Among various initiatives, the Commission’s call to strengthen the legal framework and to reinforce REACH and the CLP Regulations (Regulation (EC) 1272/2008; Regulation (EC) 1907/2006) with more coherent chemical assessment approaches and management is of special interest to the area of regulatory safety testing.

The Commission intends to extend the generic approach to risk management to ensure that consumer products including for example food contact materials, toys, childcare articles, cosmetics, detergents, furniture and textiles, do not contain chemicals that cause cancers, gene mutations, affect the reproductive and endocrine systems or are persistent and bio-accumulative. Furthermore, consumer products should be free of harmful chemicals possibly affecting the immune, nervous, or respiratory system or any other specific organ.

Implementation of this policy will require more toxicological information thus, new testing requirements that might pose a number of technical, methodological and ethical challenges. These may encompass the type and the amount of available tests needed for new enhanced safety assessments, especially for new consumer products such as cosmetic ingredients for which animal testing is banned and their development might otherwise be hampered (Regulation (EC) 1223/2009; Gustafson et al. 2020). Likewise, it is also necessary to consider the identification of ad hoc tests to cover specific (intermediate) toxicity effects other than the known apical toxicity endpoints as well as relevant toxicity information for more coherent chemical assessment approaches.

In a recent paper, we highlighted the need to make better use of toxicity studies for human health, especially for the prediction of complex systemic endpoints, and provided some examples where, integration of information across different toxicity endpoints can be explored to devise efficient testing strategies (Madia et al. 2020).

In the area of consumer products for example, the International Cooperation on Cosmetics Regulation (ICCR) has recently outlined the principles underpinning a new approach that aim to address topical and systemic toxicity by integrating information from new approach methodologies (NAMs) only, the so-called next-generation risk assessment (NGRA) (Dent et al. 2018; Baltazar et al. 2020).

Based on the above, we elaborated further on the integration of toxicity information across endpoints and we decided to approach the issue from a different perspective. By moving backward to the information generally stored in toxicological dossiers, we performed a fine dissection of available testing methods and approaches for the various toxicity endpoints. This allows the investigation of new modalities to explore and exploit the information therein. The overall aim is to explore possibilities for evaluating hazard by combining information across different systemic toxicity endpoints, rather than considering them individually, and integrating them also with different data sources. This integrated “comparative toxicology” approach can in principle be applied to any toxicity endpoint and is expected to result in a set of options for waiving redundant toxicity studies (mainly long-term ones, including carcinogenicity) with the final goal to design more efficient testing strategies amenable also to classification and labelling.

For the exercise described in this paper, we defined our scenario, carcinogenicity endpoint and, we made use of the ten key characteristics (KCs) of carcinogens (Smith et al. 2016) to build a matrix to organise the information in a structured way. This scenario was chosen as the opportunity to integrate information across systemic health endpoints that are particularly relevant to the evaluation of the carcinogenic potential of substances and, it complements with a number of ongoing activities in different sectors such as, pharmaceuticals, agrochemicals and industrial chemicals (Jacobs et al. 2020; Krewski et al. 2020; Luijten et al. 2020; Sistare et al. 2011; van der Laan et al. 2016). The study was completed with the analysis of three different publicly available toxicological dossiers for the pesticide Linuron, and industrial chemicals 1,2-dichloroethane and hydroquinone, which served as a proof of concept.

## Methodology to build a matrix for the integration of information

### Source of information

Literature search, biology, physiology, pharmacology text-books review and manual interrogation of tagged articles in PubMed (bibliographic database largely comprised of biomedical literature maintained by the US National Library of Medicine) served to identify the observed effects/parameters necessary to describe each of the ten KCs of carcinogens. Further, key sources of information for the detailed analysis of different toxicity apical endpoints were identified. Whilst selecting primary sources for collection of toxicological testing methods, the level of test method standardisation and status of validation/regulatory acceptance were also taken into account and annotated (Online Resource 1, row 6). For this purpose, all available test protocols, test guidelines and guidance documents were interrogated from:

- OECD iLibrary (https://www.oecd-ilibrary.org/). Specifically, in the OECD Guidelines for the Testing of Chemicals Section 4, about 150 of the most relevant internationally agreed testing methods, used to identify and characterise potential hazards of chemicals, are available.
- European Chemicals Agency (ECHA) and European Food Safety Authority (EFSA) Guidance Documents: e.g. (ECHA R7a 2017; ECHA EFSA Guidance 2018).
- EURL ECVAM—DB-ALM: The ECVAM Database Service on Alternative Methods to Animal experimentation (DB-ALM)—Online, which provides also the INVITTOX protocol collection, method-summary descriptions, test results, details of validation studies; (Q)SAR models, both available from the JRC Data Catalogue, https://data.jrc.ec.europa.eu/dataset?q=eurl+ecvam&sort=sort_criteria+desc.
- Standard evaluation procedures (SEPs), guideline and assay documentations developed by the U.S. Environmental Protection Agency (EPA) specific to pesticides, https://www.epa.gov/pesticide-registration/pesticide-registration-policy-and-guidance.
- Test methods reported in scientific peer review publications (e.g. PubMed, Scopus).
- US Food and Drug Administration (US FDA) Collections of Guidance Documents, https://www.fda.gov/regulatory-information/search-fda-guidance-documents.
- Guidelines from The International Council for Harmonisation of Technical Requirements for Pharmaceuticals for Human Use (ICH) and European Medicines Agency (EMA), https://www.ich.org/home.html.
- National Toxicology Program (NTP) ToxCast Data, specifically to the collection of assays and the platform sources, descriptions, and protocols used to test chemical activity in high-throughput screening, https://www.epa.gov/chemical-research/toxcast-data-generation-toxcast-assays.
- Collaborative Adverse Outcome Pathway Wiki (AOP-Wiki), primary repository of qualitative information for the international AOP development effort coordinated by the Organisation for Economic Co-operation and Development (OECD), https://aopwiki.org/.
- Exposome-Explorer: a manually-curated database on biomarkers of exposure to dietary and environmental factors from International Association for Cancer Research (IARC), http://exposome-explorer.iarc.fr/.
- Toxic Exposome Database, T3DB bioinformatics resource that combines detailed toxin data with comprehensive toxin target information (metabolomics). This project is supported by the Canadian Institutes of Health Research, Canada Foundation for Innovation, and by The Metabolomics Innovation Centre (TMIC), http://www.t3db.ca/ (Lim et al. 2010; Wishart et al. 2015).
- Carcinogenesis Vol 36. Assessing the carcinogenic potential of low-dose exposures to chemical mixtures in the environment: the challenge ahead (Carcinogenesis 2015) https://doi.org/10.1093/carcin/bgv090.
- EU Commission documentation from Directorate General Health, Scientific Committees on risk assessment methodologies and approaches (SCHER/SCCP/SCENIHR 2009).

### Collection and organization of toxicity information

To integrate the information from different sources and studies and to perform a comparative analysis across different toxicity endpoints, useful to assess carcinogenicity (Krewski et al. 2020), we mapped available information from toxicity test methods to the KCs of carcinogens. Recently described by IARC as a number of properties and modes of action by which agents contribute to carcinogenesis (Smith et al. 2016), the KCs were used here to guide and organise the toxicology information. By means of the ten KCs, we were able to deconstruct toxicity test methods (described in test guidelines or study protocols) to allow the overall information across to be organised in a systematic way. This aimed to facilitate the description and the comparative evaluation of the toxicity information and the identification of where the latter is stored. For this reason, we applied a step-wise approach to build a matrix for the collection and organisation of toxicity information.

#### Step 1. List of key characteristics of carcinogens and analysis of parameters to describe them

In a first step, we performed a curated review of literature to identify major parameters (named in Table 1 as observed effects) able to describe each of the ten KCs of carcinogens (KC1 = Act as an electrophile either directly or after metabolic activation; KC2 = be genotoxic; KC3 = alter DNA repair or cause genomic instability; KC4 = induce epigenetic alterations; KC5 = induce oxidative stress; KC6 = induce chronic inflammation; KC7 = be immunosuppressive; KC8 = modulate receptor-mediated effects; KC9 = cause immortalisation; KC10 = alter cell proliferation, cell death, or nutrient supply) thus furthering the work performed recently by Smith and colleagues (Smith et al. 2020).

**Table 1:** Observed effects defining the key characteristics of carcinogens

Key characteristics of carcinogens	Observations/observed effects	In silico	In vitro	In vivo and/or ex-vivo	Human
1. Act as an electrophile either directly or after metabolic activation	Functional group	a	a, b		
	Protein reactivity/binding	a	a	a, b	
	DNA binding/affinity	a	a, b		
	Require metabolic activation (CYP induction; aromatase inhibition; **GTS conjugates**)	a	a	a, b	
	Phospholipids binding	a	a, b		
	Phys–Chem properties	a	a	a	
2. To be genotoxic	Gene mutations	a	a	a	*
	Micronucleus (chromosome instability)	a	a	a	*
	Chromosome aberrations numerical and/or structural	a	a, b	a, b	*
	Unscheduled DNA synthesis	a		a	
	DNA adducts	a	a	a, b	*
	General DNA damage	a	a, b	a	*
	**p53** Activation/inhibition		a	a	*
	GADD45a activation/inhibition		a	a	
	**Histone gH2A **foci		a		
	Poly(ADP-ribose) polymerase induction (PADPR)				
	**H3** **phosphorylation**/induction		a		
3. Alter DNA repair or cause genomic instability	Microsatellite instability (MLH1,MSH2, MSH6, PMS2)		c	a	
	Mitotic recombination (mutation, HR- BRCA1, BRCA2, PALB2)		a		
	Mutagenic events (long-patch) by excision repair			a	
	Nonrepair, misrepair or misreplication or SNPs in DNA repair genes e.g. (RAD51, XRCC2, XRCC3, XRCC1, PARP1, MUTYH)		a	a	*
	Replication fidelity (non-homologous end joining (NHEJ): Ku70, Ku80, Xrcc4)		c		
	Apoptosis	a	a, b	a, b	
	Gross Chromosomal Rearrangements (GCRs): Chromosome and chromatid-type ab. breaks- exchanges—DSBs translocations	a	a, c	a	
	No. of polyploidy cells as indication of mitotic process inhibition		a	a	
	No. of cells with endo-reduplicated chromosomes as index of cell cycle progress inhibition		a	a	
	CpG island methylation phenotype (CiMP) (DNA hypomethylation)		a, c	c	
	Transposon activation		b, c		
	Mutations in cell cycle genes (**P53**, GADD45, CDKs)		a	c	
	Telomere shortening or dysfunction or telomerase expression modification		a, b, c	c	*
	Hyper-recombination		a, b	c	
4. Induce epigenetic alterations (consult OECD DRP 178)	DNA methylation (5-methylcytosine)		b, c	c	*
	R-loop formation				
DNA modification/ structure	DNA binding sites for transcription factors		c	c	
	Chromatin Looping (Kit regulation by Gata1/Gata2				
	Nucleosome positioning		c	c	
	Transposon activation		c	c	
	Transcriptional profile		c	c	
	Transcription factors and regulators (GATAD2A, GATAD2B, FOXO1, CEPBa, CEPBb, SMAD4 etc.)		c	c	
	Clinically validated biomarkers				*
DNA histone modification	Histone methylation		c	c	
	Histone acetylation		c	c	
	Histone phosphorylation		a, c	c	
	Transcription factors and regulators (Sirt1, Sirt2, Hdac6, Sox1, Sp1 etc.)		c	c	
RNA mediated	Modification of the expression (miRNA, siRNA, piRNA and lncRNA)		c	c	*
	Transcriptional data and alternative splicing		c	c	
	Polyamines level and function				
	Clinical biomarkers (e.g.has-miR-96)		c	c	
	RNA editing (post-transcriptional alterations, e.g., miRNA, siRNA, piRNA and lncRNA)				
5. Induce oxidative stress	8-hydroxyguanosine (8-OHG)		b	b	*
DNA/RNA damage	8-hydroxydeoxyguanosine (8-OHdG)		a	b	*
	Abasic (AP) sites			b	
	BPDE (benzo[a]pyrene diol epoxide) or other epoxides DNA Adduct		a, b	a, b	*
	Double-strand DNA breaks or SSB also through ODC induction	a	a	a	
	General DNA damage (e.g. Comet with FPG)	a	a, b	a, b	
	Mitochondrial DNA damage		a, b	b	
	DNA mutations (Ames test with TA102)		a		
	UV DNA damage (CPD, 6-4PP) also Ames test with TA102		a		
Lipid peroxidation	4-Hydroxynonenal (4-HNE)				
	Malondialdehyde (MDA) or Thiobarbituric acid reactive subst. (TBARS)			b	
	Oxidized low-density lipoprotein (LDL)				
	Hydroxyoctadecadienoic acid (HODE)				
	Advanced lipoxidation end products (ALE)	a			
	Oxysterol				
	HOC1-modified LDL				
	Ornithine Decarboxylase (ODC) induction		c		
Arachidonic-acid-derived oxidation	8-iso-Prostaglandin F2alpha (8-isoprostane)				
	Isofurans				
	Isolevuglandinins				
Protein oxidation/nitration	Protein Carbonyl Content (PCC)				
	Amino acid oxidation (e.g. 3-Nitrotyrosine, 3-chlorotyrosine, dityrosine, carboxymethyl lysine, cysteine/cysteine, homocysteine/homocysteine)		c		
	Advanced Glycation End Products (AGE)		a	a, b	*
	Advanced Oxidation Protein Products (AOPP)				*
	Tryptophan adducts				*
	BPDE (benzo[a]pyrene diol epoxide) protein adduct				*
ROS/RNS	Universal **ROS** / RNS		a		
	Hydrogen peroxide		a		
	Superoxide /singlet oxygen		a		
	Nitric oxide				
Antioxidants levels or activity	Catalase		c		SNP
	**Glutathione** activity // **GSTs conjugates** // detoxification		a, c		SNP
	Superoxide dismutase		a, c		SNP
	Thioredoxin		c		SNP
	Aryl-esterase/paraoxonase (PON)		c		SNP
	OSI (ratio of total antioxidant capacity/total oxidant status)				
	Oxygen Radical Antioxidant Capacity (ORAC)				
	Hydroxyl Radical Antioxidant Capacity (HORAC)				
	Total Antioxidant Capacity (TAC)				
	Induction of "antioxidant genes" (e.g.** SRXN1**)		a, c	c	
	Cell-based exogenous antioxidant assay				
	**Mitochondrial activity and function**		a, c		*
	**ER stress response** -TFs—gene expression specific, **OXOPHOS, Hsp70 chaperones**		a, c	a, c	
	ARE early response—TFs (e.g. **NRF2** or **SRNX1, HMOX1**)		a, c	c	SNP
6. Induce chronic inflammation	Acute/subacute inflammatory infiltrate (acinar and/or interstitial) histopathology oestrogenic compounds effect in prostate		a, b	a, b	
	Chronic infiltrate of lymphocytes +neutrophils, with poss. macrophages—liver		a	a, b	
	Detection of different cellular types immunohistochemistry		a	a, b	
	Enzyme induction e.g. **CYP2E1, GSTM1, GSTT1, GSTP1**	a	a, b, c	a	SNP
	Blood/serum test: clinical biochemistry // urinalysis			a	
	Delayed-type hypersensitivity	a	b	a	
	Serum amyloid A (SAA)			b	
	Serum C-reactive protein (hsCRP), interleukin (IL)-6,** tumour necrosis factor (TNF)-α**, **IL-8**, soluble (sTNFR) I and II		b	b	*
	Angiogenesis (**VEGFA**, bFGF, HGF)		c	a	
	Early response activation monocytes- cytokines cell surface		a, b, c	a, b	
	Cyclooxygenase (e.g. **COX1**, COX2, 11-hydroxyeicosatetraenoic acid (HETE))		c	c	
	Pro-inflammatory cytokines (IL-6,** IL-8** IL-1b, IL-18, IL-17,** (TNF)-a**)		a, b, c	a, c	
	MHC class II (lymphocytes activation)		a, c	c	
	Pro-inflammatory **ROS** and RNS		a, b, c	b	
	Endocrine-driven deregulation of immune system (hypothalamic-pituitary-adrenal (HPA) axis)		a, b, c	a, b	
	Clinical signs (General Appearance, Skin and fur, Eyes Nose, Mouth, and Head, Respiration, Urine, Faeces, Locomotor, FOB)		a	a, b	
	Organ weight (e.g., liver, kidney, thymus, adrenal gland) // gross pathology	a		a, b	
	Body weight/food consumption			a, b	
	Early response—Transcription Factors (**HIF-1α**, HIF-2α, **NF-κB, CREB**, STAT family)		a, b, c	c	
7. Be immunosuppressive	Total and absolute differential leukocyte counts—in serum			a, b	
	Globulin levels and A/G ratios			a, b	
	Lymphoid organs / tissues histopathology e.g. Spleen, thymus ,lymph nodes			a, b	
	T-cell dependent Ab response—blood			a	
	Identification/count of leukocyte subsets using antibodies FC			a	*
	NK cell activity		c	a	*
	Host resistance to bacteria, virus, tumour cells			a, b	
	IC50—immune-suppression on total white blood cells		a		
	Lymphocytes/CD count and characterization—IC50 immunosuppression		a, b	a, b	
	Macrophage/Neutrophil funct: phagocytic, oxidative burst, chemotaxis, cytolytic		a		
	Cytokines release (IL-17, IL-10, TGF-β, PGE2)		a, c	c	
	Bone marrow proliferation (myelotoxicity)—test also in CD71 cells		a	a, b	
8. Modulate receptor-mediated effects	Receptor binding (XRs: CAR, PXR,** AhR**; NRs: thyroid hormone receptor, retinoic acid receptor, AR, ER, RXR, FGFR, **PGC1a**)	a	a, b, c	b	
	Transactivation ER or AR //	c	a, c	c	
	Hormonal serum level ( e.g., T3,T4, TSH)			a, b	*
	Enzyme activity (also **CYP** and **LDH**)	a	a, b, c	b	*
	Hormone production E2 or Testosterone	a	a		
	Effects on corticosteroid synthesis	a	a, b, c	c	
	ACTH corticotropin		a, c	b, c	
	LH and chorionic gonadotropin GC		b, c	b, c	
	Vitamin D		b, c	b, c	
	Vitamin A			b, c	
	CYP induction	a	a, c	a, c	
	GRs glucocorticoid receptor	a, c	a, b, c		
	Progesterone receptor PR	a	c		
	IGF-1 gene expression or GH		a, c	c	
	AVP vasopressin		c		
	Organ weight (e.g., uterus, testes, prostate, epididymis, adrenals) Hypertrophy?			a, b	
	Cholesterol level HDL/LDL ratio			a, b	
	Endocrine responsive organs/tissues—Histopathology—Reproductive functions		a	a, b	
9. Immortalization	LOH: Loss of heterozygosity		c	a, c	
	Dysfunction of p-53 cascade: deregulation of cell-cycle genes (e.g. p14, MDM2, p33)		a, c	b, c	
	Dysfunction of p16 or p27		c	c	
	Loss of function senescence genes: 1, 4, 6, 7, 11, 13, 17, 18 and X Chr				
	Loss of balance of +/− regulators of growth			b	
	Alternative mechanism for lengthening of telomeres (ALT)		c	c	
	Increased telomerase activity: no shortening of telomeres		b, c	c	*
10a. Alter cell proliferation	Chemotaxis		a	a, b	
	Haptotaxis				
	Transmigration		a	a, b	
	Proliferation (e.g. mitotic indexes, Dysregulation in signalling pathways: MAPK , Rec. tyrosine kinase, EGF)		a, c	a, b	
	Alpha 2u-globulin accumulation (kidney)				
	Altered cell morphology // Cell Transformation // Foci	c	a, c	a, b	
	Differentiation (EMT, Wnt, TGF-b, Notch)		a, c	c	
	BrdU labelling // DNA synthesis increase			a, b	
	PCNA labelling				
	**Nuclear size**		a		
	Ornithine decarboxylase induction		c	c	
	Hyperplasia (increase number of cells)			a, b	
	Inflammation/regeneration		a	a, b	
	Loss of contact inhibition (e.g. Hippo pathway)		a, c	c	
	Cell cycle regulators (Cyclin-dependent kinases, p16, p21, p53, G1 arrest, G2 arrest)		a, c	b, c	
	Angiogenesis (VEGFA, bFGF, HGF)	a	a, c	a, b, c	
	Morphology—Cell to Cell communication—Gap junctions		a, b, c	a, b	
	Neoplastic lesions- tumour			a, b	
	Hypertrophy (organ weight) - Regeneration (e.g., liver)			a, b	
	Invasion (**EMT, MMP2 and MMP9 expression, invadopodia**)		a, b, c	a, b, c	
10b. Cell death, or nutrient supply	Cytotoxicity		a, b, c	a, b	
	**Cell cycle markers** (e.g. **pH3**, mitotic index)	a	a, b, c	a, c	
	**Apoptosis **(assays: TUNEL, DNA laddering/DNA fragmentation, Apoptotic nuclei, Annexin-V (membrane integrity), Bcl-2/Bax ratio, **Caspase-9, Mitochondrial membrane**)		a, c	a, b	
	Anti-apoptotic members (BCL-2, BCL-XL, BCL-W, MCL-1 and A1/BFL-1)	a	a, c	c	
	Autophagy markers, mt-function		a, c		
	Cell energetics and metabolism (**ATP consumption, OCR oxygen consumption rate, PGC1a, PPARg**)		a, b, c	b, c	
	Necrosis		a, b	a, b	

Major descriptors of the key characteristics of carcinogens. These represent the observed effects/observations that were mapped across different model systems (in silico*, *in vitro*, *in vivo and/or ex-vivo) and define the key characteristics. Though partly redundant, some of the observed effects appear under different key characteristics since the physiological processes they are involved in are interlinked

*a, b, c* categories used to organise the observed effects in the matrix: **a** indicated in the test protocol or in the test guideline as a specific endpoint result; **b** embedded within the test protocol or the test guideline documents as a part of the information needed to obtain specific results or extrapolated in an indirect manner; **c** derived from recent test protocols and/or investigative research studies

*, SNP observed effects reported as human markers of exposure/disease and relevant single-nucleotide polymorphism (SNP)

In bold: markers that are considered in the cell stress panel proposed by Baltazar et al. 2020 and Hatherell et al. 2020

Details on the collection, source of information and distribution of the observed effects are reported in “Methodology to build a matrix for the integration of information” of this manuscript and in Online Resource 1

The review and initial grouping of observed effects was based on biological and toxicological knowledge and it was initially performed without taking into consideration the test methods, the test systems or test models used to detect them. This facilitated inclusion of information while avoiding bias e.g. linked to accuracy/level of detection. As such, Step 1 served to build the phenotypic anchoring useful to organise the information, to first identify major mechanisms and to highlight, whenever available, relevant biomarkers of effects.

The observed effects were reported and grouped for each single key characteristic as shown in Table 1.

#### Step 2. Selection of relevant test methods across different toxicity endpoints

In a second step, we sought and included known available toxicity studies and protocols that might be able to measure the observed effects collected for each single KC. It was possible to include the information from various test methods, regardless of the test system used (i.e., in silico, in vitro, ex vivo, in vivo)*,* as no specific sorting was applied in the previous step.

Although not exhaustive, the collection of methods included test guidelines documents (e.g. from OECD, EPA, FDA), validated test methods or methods undergoing validation (e.g. from EURL ECVAM, DB-ALM collection) and those recently recommended by specific regulatory authorities. Finally, we included more recent test methods under investigation and published in peer review papers.

We reported the type of information that each single method provides. The information was then organised according to the observed effects described at Step 1.

**Fig. 1:**
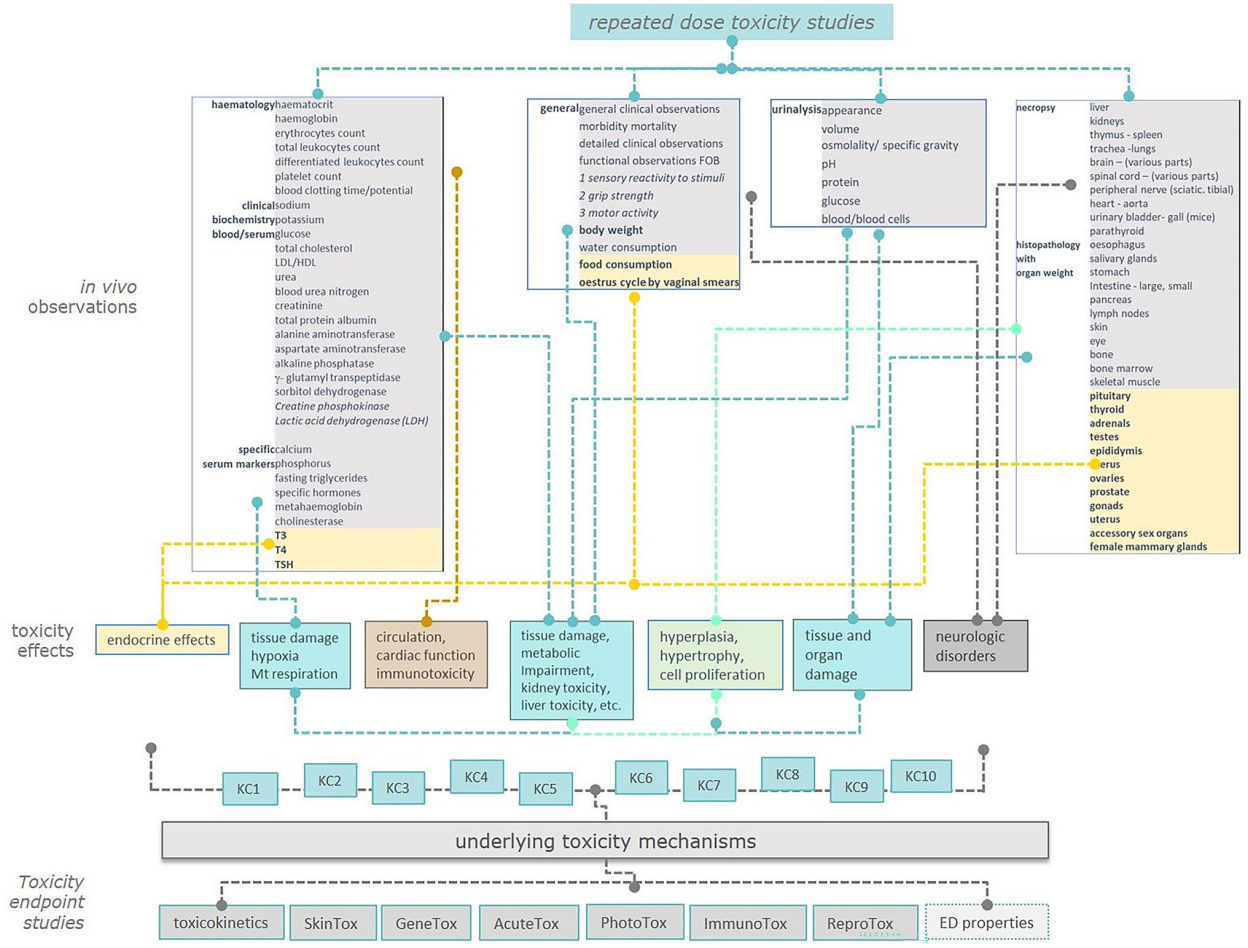
Analysis of repeated dose toxicity studies. The study protocols and test guideline documents (e.g., OECD test guidelines TG 407, 408, 451 and similar) are deconstructed to extrapolate relevant information. In vivo observations (from the annexed tables for test report of results) are linked to major toxicity effects and then mapped to the observed effects of the KCs of carcinogens (KC1-KC10) reported in Table 1. Finally, they are linked to possible underlying mechanisms which can be also shared across other toxicity endpoints

Within the description of test methods and central to this phase, we initially performed a fine dissection of acute systemic toxicity study protocols and of repeated dose toxicity (RDT) studies: sub-acute, sub-chronic, and chronic and, the 2-year-rodent bioassay (Fig. 1 and Online Resource 1). The focus here was to extrapolate the embedded information from in vivo systemic toxicity studies in terms of types of analysis (e.g. clinical biochemistry, urinalysis and histopathology), endpoints measured and any biomarker available, and to align the information to the observed effects describing the ten KCs. For this purpose, we based our approach on the “Mode of Action Framework” concept and made use of the recommendations reported in the OECD guidance document 116 that aimed to guide the optimisation of long-term toxicity studies design (OECD GD 116 2014). This helped the identification of information relevant to the mechanistic understanding of toxicity.

#### Step 3. Crossing of information: observed effects to guide the mapping of test methods information relevant to cancer

The approach used in step 2 was finally extended to the description of available test methods (in silico, in vitro, ex vivo, in vivo) for other toxicity endpoints such as, skin sensitisation, genotoxicity, phototoxicity, immunotoxicity, etc. This was done to analyse how data collected from other endpoints would inform the ten KCs of carcinogens (Online Resource 1). The approach was elaborated on the protocol for hazard identification proposed by Schwarzman and colleagues on the screening for chemical contributions to breast cancer risk (Schwarzman et al. 2015).

#### Step 4. Further analysis: comparative analysis, gaps, overlaps, limiting factors

This step included a comparative analysis across different toxicity tests with the aim of identifying gaps, overlaps or limiting factors. Also, we investigated opportunities to enrich data collection with information provided by other databases reporting on human exposure data or information on specific biomarkers. For this purpose, we expanded the matrix by including epigenetic information (based on literature review), data from the EPA ToxCast program (Chiu et al. 2018), QSAR models and AOPs. To include human relevant information, we also aligned to the KCs of carcinogens biomarkers of disease (Carcinogenesis 2015) and SNPs (single-nucleotide polymorphisms) and susceptibility risk factors for disease (as reviewed by SCHER/SCCP/SCENIH 2009; Costa et al. 2019) (Online Resource 1).

### Data annotation

To perform a qualitative evaluation of available information, data collected across various test methods, in vitro, in silico or in vivo observations, were annotated and grouped. There were three main categories of observed effects, depending whether the observation was:

- a. indicated in the test protocol or in the test guideline as a specific endpoint result (e.g., gene mutation measured in the Ames test, highlighted in red, Online Resource 1);
- b. embedded within the test protocol or the test guideline documents as a part of the information needed to obtain specific results or it could be extrapolated in an indirect manner (e.g., serum analysis of blood cell populations in the micronucleus (MN) in vivo, highlighted in yellow, Online Resource 1);
- c. derived from recent test protocols and/or investigative studies (e.g., data results from ToxCast assays, highlighted in dark green, Online Resource 1).
- The grouping and categorisation of results served to track the origin of information, to further elaborate on the potential of each study protocol, finally to evaluate the level of standardisation of currently available information.

## Distribution of the information across toxicity endpoints

### Description of the matrix

The collection of observations has generated a heterogeneous dataset (Online Resource 1) that considers all three categories described above, and where scientific knowledge is mapped across the various toxicity endpoints and the ten KCs of carcinogens, regardless of the level of standardisation of test methods and systems used or, biological level of organisation (i.e., molecular, cell, tissue or organism).

**Fig. 2:**
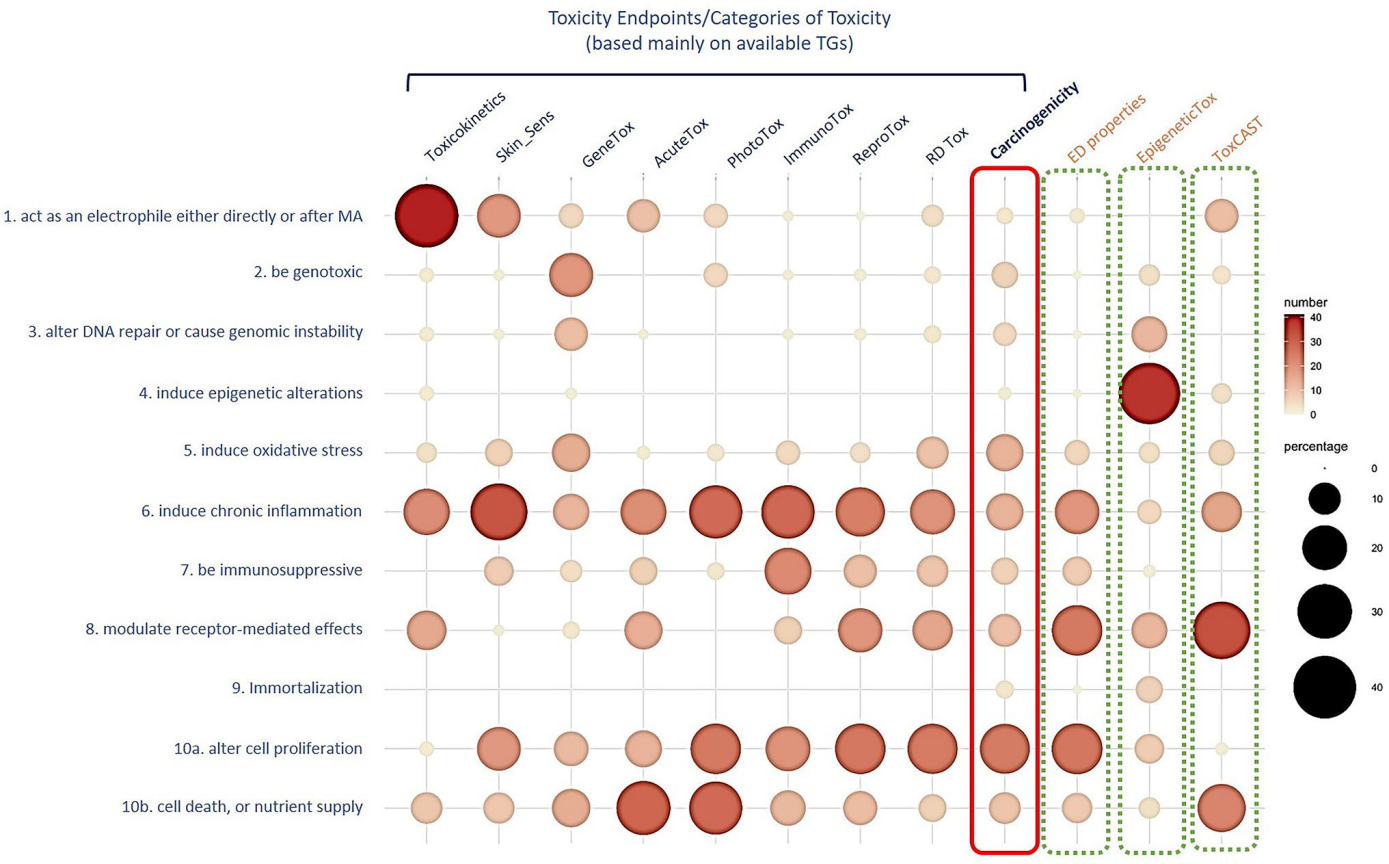
Distribution of information. Differential contribution, reported as percentage, of each toxicity endpoint to the properties (key characteristics) of carcinogens, in terms of provided information. Each regulatory toxicity endpoint can be assessed through a number of different types of assays in silico, in vitro or in vivo and/or ex vivo as reported in the Online Resource 1. Reported information is normalised (percent ratio) over the number of studies available for each single endpoint (each column adds up to 100%). Available toxicity studies for carcinogenicity endpoint (red dotted line) were also organised on the basis of KCs. Standard toxicological information can be also enriched with parameters (observed effects) derived from more recent test protocols and/or investigative studies such as those (green dotted line) describing ED properties, epigenetic alterations (EpigeneticTox) or toxicity effects detected with high-throughput-screening methodology (ToxCast data). Percent ratio for ToxCast data was calculated on the basis of selected assays as reported by Chiu et al. 2018

As such, the existing knowledge is organised, irrespective of specific indications on the regulatory use of each test method, and provides an indication of the distribution of available information. This allows to establish the differential contribution to the properties of carcinogens of the different toxicity endpoints, mainly in terms of mechanistic toxicity information (Fig. 2). The observed effects are parameters (biomarkers, enzyme activities, final test results) that define the KCs of carcinogens and describe the different toxicity effects.

As shown in Fig. 2, the endpoints toxicokinetics, skin sensitisation, genotoxicity, acute systemic toxicity, phototoxicity, immunotoxicity, toxicity to reproduction, repeated dose toxicity and carcinogenicity, are the major components in any toxicological regulatory dossier (e.g., ICH S4 2000; ICH S8 2006; ICH S1 2012; ICH S2(R1) 2012; ECHA R7a 2017), and contribute differently to the KCs of carcinogens. Notably, endocrine disrupting properties, despite not being a toxicity endpoint per se but a specific mode of action leading to various toxicity endpoints, have also been considered since, based on the criteria outlined in Commission Regulations (Regulation (EU) 2017/2100; Regulation (EU) 2018/605), specific tests have been proposed recently for the identification of endocrine disruptors in the context of EU Regulations for plant protection and biocidal products (Regulation (EC) 1107/2009; Regulation (EU) 528/2012; ECHA EFSA Guidance 2018).

Figure 2 shows that when considering the sources of information available to identify each toxicity endpoint, there is the possibility to cover more than one KC. For example, examining available studies that describe the toxicokinetics (TK) of a chemical, we identified a number of protocols, as described in OECD TG 417 (OECD 417 2010) and more recent ones, including various in vitro test systems (> 10 protocols). These studies are not only able to measure absorption, distribution, metabolism and excretion (ADME) properties but also parameters that can be related to the KCs of carcinogens, such as: act as an electrophile either directly or after metabolic activation; modulate receptor-mediated effects; induce oxidative stress; induce chronic inflammation. In addition, TK information applicable to these KCs, can be also predicted by means of physiologically-based kinetic (PBK) models. An in-house review of PBK models developed in the past 10 years (2009–2019) ((Lu et al. 2016); the WUR University (NL) collection; PubMed), has indeed provided a number of models designed to describe drug-drug interaction (DDI) or drug- or chemical-response analysis, distribution in target tissues or chemical carcinogens exposure analysis whose predictions are applicable to different KCs (Fig. 2 and Online Resource 1).

The introduction of PBK modelling in the matrix can help to predict systemic exposure from external exposures but also to integrate the information across various test methods along the KCs. Moxon and colleagues have recently described the application of PBK modelling to the NGRA based exclusively on NAMs for dermally applied consumer products and were able to provide conservative estimate of the maximal blood concentration (Cmax) for three case studies (Moxon et al. 2020).

Information sources available for skin sensitisation (7 in vitro studies, 4 different in vivo studies, QSAR models and available AOPs) may contribute to the description of many KCs ( i.e., act as an electrophile either directly or after metabolic activation, induce chronic inflammation, cell proliferation and cell death) except, induce epigenetic alterations and immortalisation.

Likewise, in the case of genotoxicity, the information derived from in vitro studies (*n* = 11), in vivo studies (*n* = 9), scrutinised so far, together with available QSAR models and AOPs may cover almost all the KCs of carcinogens, except immortalisation as reported in Online Resource 1. Certainly, all the assays aimed at the identification of the genotoxicity endpoint contribute mainly to the characteristics of: being genotoxic and alter DNA repair or cause genomic instability.

The group of available test methods (approximately, 10 protocols) in use for testing immunotoxicity contributes instead only to some of the KCs, being mainly specific to: induce chronic inflammation, be immunosuppressive and cell proliferation and cell death. It is worth noting that the majority of immunotoxicity studies are mainly recommended in safety guidelines for pharmaceuticals (FDA 2006; ICH S8 2006). This does not exclude that parameters related to the immune system are evaluated through several toxicity studies across different toxicity endpoints, as detailed in Online Resource 1.

As previously reported, the mechanistic knowledge derived by literature search and collected for acute systemic toxicity is a valuable starting-point to inform other adverse outcomes (Madia et al. 2020; Prieto and Graepel 2018). In the context of this exercise, it is possible to evaluate the extent to which such mechanisms could play a role after repeated dose exposure scenarios and eventually inform the KCs of carcinogens. Thus, the information derived from in vitro (*n* = 4) and in vivo studies (*n* = 7), QSAR models and available assays from the ToxCast program (EPA) may contribute to many of the KCs of carcinogens (Fig. 2). While the major contribution is for induce chronic inflammation and cell proliferation, cell death and nutrient supply KCs, the group of acute systemic toxicity tests can also inform: act as an electrophile either directly or after metabolic activation, induce oxidative stress, induce chronic inflammation, be immunosuppressive and, modulate receptor-mediated effects.

Similarly, the five protocols for the identification of phototoxicity hazard contribute mainly to induce chronic inflammation and cell proliferation, cell death and nutrient supply KCs. However, by exploiting their potential, some of these protocols can also inform: act as an electrophile either directly or after metabolic activation, to be genotoxic and induce oxidative stress.

Notably, by deconstructing available test methods and approaches for toxicity to reproduction and target organ toxicity after repeated exposures (mainly in vivo studies), we were able to identify a number of observed effects (in vivo observations). Studies available for both categories of toxicity are highly informative for the KCs of carcinogens induce chronic inflammation, be immunosuppressive, and modulate receptor-mediated effects and cell proliferation, cell death and nutrient supply. The overall pattern of information contributing to these KCs is substantially similar between the two toxicity endpoints.

The grouping of test methods scrutinised so far for the above categories of toxicity has enabled to describe in a qualitative dimension the distribution of information (Fig. 2). It also enabled to identify major contributors to the knowledge of the carcinogenic potential of substances but also areas of consistent lack of knowledge in terms of observed effects and hence available assays.

For example, KCs describing major mechanisms involved in toxicity outcomes such as oxidative stress, chronic inflammation, and alterations in cell growth can be detected by means of different test methods and test systems. They are routinely evaluated in a number of studies from in silico to in vitro and to in vivo. Instead, alter DNA repair or cause genomic instability, induce epigenetic alterations and immortalisation are still not fully incorporated within available regulatory toxicity studies and rarely investigated, despite their key role in carcinogenesis. In agreement with our observations, Krewski and colleagues (Krewski et al. 2020), analysing the KCs associated with 86 Group 1 human carcinogens reviewed by IARC, reported that information on epigenetic alterations derives mainly from human studies, both in vitro and in vivo, mostly epidemiological investigations. For alter DNA repair or cause genomic instability, epigenetic alterations and immortalisation investigations, a conspicuous number of assays and methodologies are available and in use routinely in the research field. However, as for other applications, i.e. new methodologies and “omics” techniques which are currently shaping cancer biology research (Nature various 2020), are not applied yet on a routine base in the regulatory context.

### Mechanistic information provided by in vivo studies and new approach methodologies

As summarised in Fig. 2, the matrix built over the collection of observed effects allows the alignment of toxicity information in terms of mechanistic knowledge provided by each single study. This helps to visualise where relevant information is stored and how it can be shared across different toxicity endpoints and more importantly, whether it can be used to inform one toxicity endpoint from another (Madia et al. 2020).

**Fig. 3:**
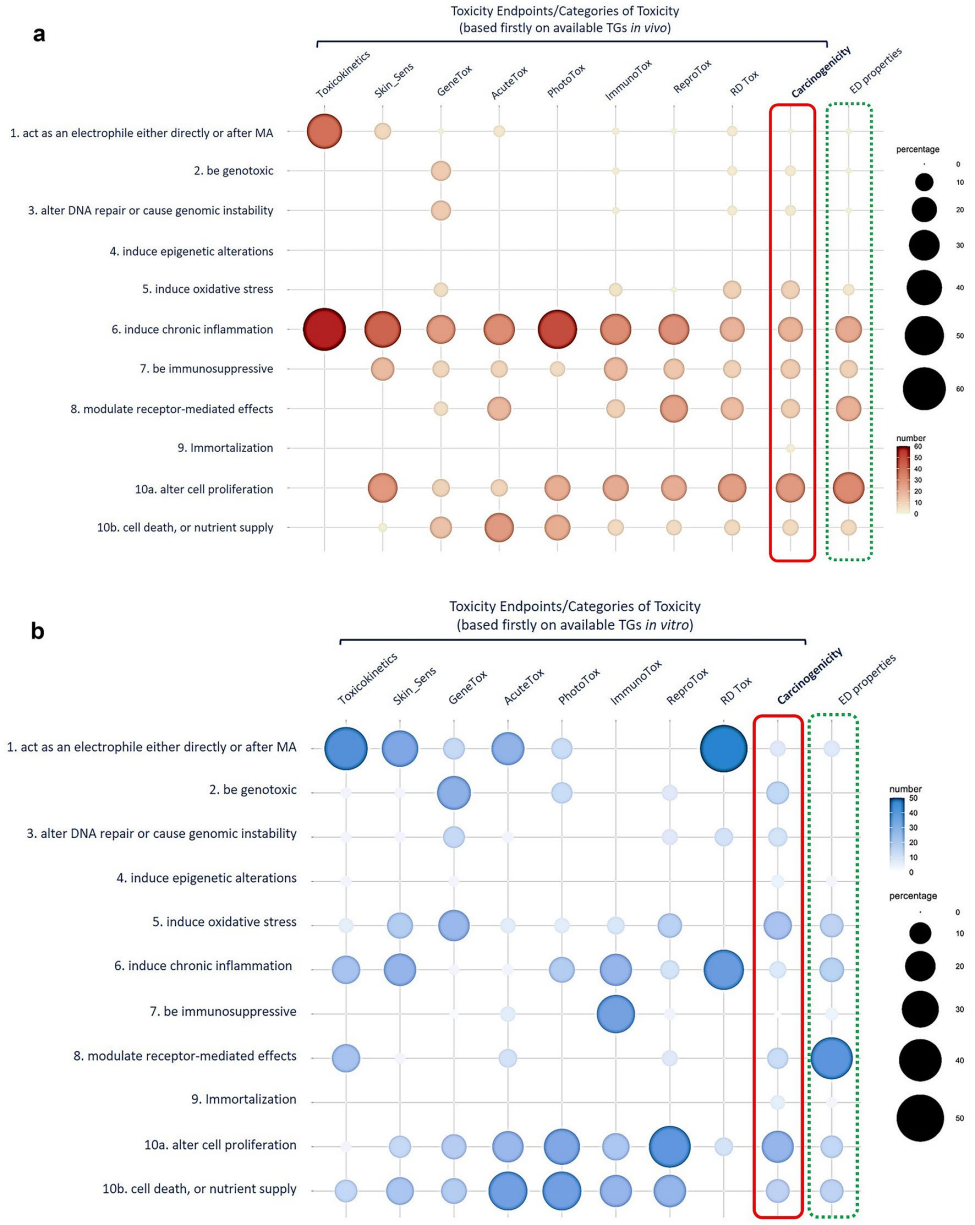
In vivo studies and NAMs-based contribution to toxicity information. Differential contribution, reported as percentage, of each toxicity endpoint to the properties of carcinogens, in terms of provided information either from in vivo studies (**a**) or from NAMs (**b**). The relative contribution to the ten key characteristics per each single endpoint changed according to the number of studies available. Available toxicity studies, in vivo and NAMs, for carcinogenicity endpoint (red dotted line) and those (green dotted line) describing ED properties (ECHA EFSA Guidance, 2018) were also organised on the basis of KCs

A number of parameters can be observed and are included per single in vivo study: general clinical observations, food consumption, toxicokinetic data, clinical biochemistry parameters, histopathology, ideally performed on every single organ, urinalysis, and/or other specific parameters as macroscopic developmental and reproductive effects, depending on the study endpoint and relative study design (Online Resource 1). The majority of these observations, even if not mechanistic per se, can be used to derive mechanistic information based on evidence and to define the KCs of carcinogens. However, most of the toxicity information provided by in vivo studies across different endpoints is highly redundant. The in vivo studies reported in the analysis (Online Resource 1) repeatedly inform some of the KCs of carcinogens, in particular inflammation, immune-response, receptor-mediated effects, cell proliferation, cell death and nutrient supply (Fig. 3a). However, these are mainly defined by the following observations: acute/subacute inflammatory infiltrate (acinar and/or interstitial) from histopathology; blood/serum clinical biochemistry data, including total and absolute differential leukocyte counts in serum, urinalysis, body and organ weight, clinical signs and food consumption, tissue/cell proliferation, hyperplasia, hypertrophy, cytotoxicity, necrosis from histopathology (Online Resource 1).

Mechanistic studies, mainly in vitro, include fewer observations per single study but they can inform multiple KCs, thus reducing redundancy of information (Fig. 3b). In this case, mechanisms and specific key events at the molecular level can be investigated to provide a detailed understanding of the toxicological mode of action (Malarkey and Hoenerhoff 2013) that conventional in vivo studies may not provide. Derived information is more heterogeneous than that derived from the in vivo counterpart. The observed effects reviewed so far and included in Table 1 are highly redundant and NAMs capable to identify them are not all in place within the regulatory context. Nevertheless, NAMs included in this first exercise show the opportunity to enrich mechanistic information across multiple KCs and multiple toxicity endpoints. This is for example the case of specific chemical properties or known key molecular players, i.e., transcription factors, regulators, mediators of effects whose function relates to different KCs and inform different endpoints (the example of the Nrf2-Keap1-ARE signalling pathway is detailed in Box 1). Furthermore, they may be equally described by means of in vitro, in silico*,* or more recently developed *‘omics’* approaches applied to different endpoints (Online Resource 1). Along these lines, Baltazar and colleagues recently illustrated the application of NGRA for the safety assessment of systemic toxicity of cosmetic products to a case study (coumarin) that included the use of integrated information across different toxicity endpoints and by means of various methodologies: information predicted from PBK models and in silico alerts, data from genotoxicity studies such as the Toxtracker (Hendriks et al. 2016) test method, cell stress panel, and high-throughput transcriptomics (HTTr) (Baltazar et al. 2020).

The matrix built over the observed effects can serve as a repository and a guide to identify information relevant to the properties of carcinogens. This gives the opportunity, on the basis of a mechanistic read across, to select available ad hoc test methods that can be used to avoid redundancy of testing but also to identify where relevant information is missing. This is shown in the matrix, as mentioned above, for the KCs of induce epigenetic alterations and immortalisation. Also, in the case of the KC to be immunosuppressive, the number of available test methods is limited in their use and application and are not yet sufficient to cover specific mechanisms of immunosuppression (Online Resource 1 and Fig. 2 and Fig. 3).

**Box 1. Nrf2-Keap1-ARE signalling pathway role across toxicity endpoints**

- Skin sensitisers, particularly cysteine-reactive skin sensitisers, have been shown to induce protective genes regulated by Nrf2-Keap1-ARE regulatory pathway (Kleinstreuer et al. 2018). The Keratinosens test method (OECD 442D 2018) is based on this principle. Similarly, the Sens-is test method is proposed to monitor the expression of a panel of 65 genes, including NRF2 in Reconstructed human Epidermis (RhE) for irritancy and sensitisation (Cottrez et al. 2015).
- The Nrf2 signalling pathway represents one of the main cell defence mechanisms (Leinonen et al. 2014; Basak et al. 2017) and is considered a master regulator of redox homeostasis. It has been shown to play a role in different neurodegenerative diseases, aging, diabetes, photo-oxidative stress, cardiovascular disease, inflammation, pulmonary fibrosis, acute pulmonary injury, and also cancer (Kansanen et al. 2013; Jaramillo and Zhang 2013).
- The NrF2-Keap1 transcriptional activation has been reported to be elicited in response to liver and kidney toxicants (Limonciel et al. 2018; Xu et al. 2019). As such, the activation of the Nrf2 response is relevant to skin sensitisation as well as other toxicity endpoints as genotoxicity, acute toxicity and/or repeated dose toxicity, and carcinogenicity.
- In vitro genotoxicity tests include as well directly or indirectly the analysis of Nrf2-Keap1-ARE regulatory pathway (e.g. GreenScreen, Toxtracker, DNA multiflow). In the Toxtracker test method, for example, the Nrf2 signalling activation is determined to investigate whether oxidative stress may contribute to the genotoxic and cytotoxicity profile of a compound (Hendriks et al. 2016).
- The Nfr2 transcription factor is one of the 36 biomarkers included in the cellular stress panel proposed as part of the next generation risk assessment (NGRA) approach for systemic toxicity testing designed for cosmetic ingredients by Hatherell and colleagues (Hatherell et al. 2020).

### Organising the toxicity information for three substances into the matrix

To evaluate whether it is possible to organise toxicity information as shown in Fig. 2 and Fig. 3 in a real scenario, we populated the matrix with the information provided in publicly available toxicological dossiers for three rich-data substances. We chose the plant protection product Linuron and two industrial chemicals, Hydroquinone and 1,2-dichloroethane. These substances have also been used to elaborate on the concept of cross endpoint evaluation (Madia et al 2020).

#### Linuron

Linuron (CAS no.: 330-55-2) is a herbicide, with harmonised classification as possibly carcinogenic (cat. 2) and toxic for reproduction (cat. 1B) in accordance with CLP Regulation (Regulation (EC) 1272/2008). According to the Final Renewal Report Commission Staff Working Document, Linuron is considered to have endocrine disrupting properties in accordance with Annex II to Plant Protection Products Regulation (Regulation (EC) 1107/2009). Information available for Linuron was extracted from EFSA Draft Assessment Report (DAR) and Renewal Assessment Report (RAR) (EFSA pesticides Dossiers 2020).

As reported in the DAR, a number of studies considered for the final evaluation were quite old and several results were conclusive but not sufficient for classification. Some of the most recent studies instead included in the RAR and reporting toxicology and metabolism data, were proprietary information and, as such not disclosed. Approximately more than 30 toxicity studies, regarded as valid on the base of data and experimental design quality, were summarised in the report. These were reported in the matrix and the information provided from each single study was aligned to the KCs of carcinogens (Online Resource 2).

Induce chronic inflammation, alter cell proliferation and alter nutrient supply and cell death, were confirmed to be the most investigated KCs across various toxicity studies.

The repeated dose toxicity studies (five in total) were very informative, they were performed under GLP guidelines and with a good data reporting. However, the 72.5% of the "type information" provided, was the same across the different repeated dose toxicity studies and was related to similar observed effects. Neither ED properties nor immunotoxicity conventional studies were performed.

It is worth noting that a large part of standard TGs were not filled within the table, since this information was not detailed in the DAR.

As from DAR summary evaluation: Linuron undergoes metabolic activation, is not genotoxic, induces oxidative stress and protein reaction (methaemoglobin), inflammatory response, cell proliferation, cell death (cytotoxicity) and toxicity effects to reproduction, derived by two studies (OECD 416 2001; OECD 414 2018). However, detailed mechanistic information able to describe potential receptor-mediated effects, specific to androgenic effects, and details on the carcinogenic potential was provided mainly by several additional supplementary studies both in vitro and in vivo. Interestingly, such additional studies despite not being standard studies (i.e., no TGs available) and not being performed under GLP, provided a more diverse and less redundant pattern of information (in terms of different observed effects) as compared to repeated dose toxicity studies for two specific KCs such as receptor-mediated effects and alter cell proliferation, cell death and nutrient supply, thus enriching toxicity information from 1 to 2 fold (Online Resource 2).

#### 1,2-dichloroethane

1,2-dichloroethane (CAS no.: 107-06-2) is an industrial chemical. According to CLP Regulation (Regulation (EC) 1272/2008), this substance may cause cancer (cat. 1B), is harmful if swallowed, causes serious eye irritation, causes skin irritation and may cause respiratory irritation.

The information for 1,2 dichloroethane derived from ECHA registration dossier (C&L Inventory 2020) (last modified 16th April 2019).

In the ECHA database, about 65 study reports for 1,2-dichloroethane were included. Several studies did not fulfil completely the requirements from internationally accepted guidelines or were not fully reported. For this reason, only key studies with a score of reliability of 1 or 2^1^ (ECHA R4 2011) were considered (a total of 13 studies) (Online Resource 3).

For this chemical, genotoxicity was the most informative and studied toxicity endpoint. Moreover, the information reported aligned with several observed effects describing the 10 KCs of carcinogens.

Particularly, the study reported as DNA damage [Comet], performed under GLP, was fully detailed and information-rich. The study included also a number of observations not strictly related to the standard OECD TG. Interestingly, the comet assay was performed also on mammary gland tissue. Very little information instead, was reported for the repeated dose toxicity study, despite the complexity of the study protocol.

The majority of mechanistic and informative data derived from toxicity studies performed in compliance with GLP procedures but not following any official test guideline (OECD TG). This was also the case for a specific cancer study (key study 3) investigating in detail 1,2-dichloroethane carcinogenic effects on mammary gland tissue after inhalation exposure. This resulted as the most informative study among those reported in the dossier and specifically more informative that the two conventional cancer studies also reported. Observed effects provided by the study covered almost all the KCs of carcinogens and included information on exposure markers linked to DNA damage. Among others, study parameters measured included cage side and clinical observations, feed consumption, body weights/body weight gains, oestrous evaluations, serum prolactin levels, measurement of reduced (GSH) and oxidised (GSSG) glutathione, DCE-glutathione conjugates S-(2-Hydroxyethyl)glutathione hydrochloride (HESG) and S,S’-Ethylene-bis glutathione (EBG), DNA adducts, 8-Hydroxy-2′-deoxyguanosine (8-OH dG) and S-(2-guanylethyl) glutathione (GEG) in mammary and liver tissue, Comet assay (mammary tissue), morphometric evaluation of mammary gland structure, cell proliferation (Ki-67), and histopathology (mammary tissue).

#### Hydroquinone

Hydroquinone (CAS no.: 123-31-9) is an industrial chemical. According to CLP Regulation (Regulation (EC) 1272/2008), this substance is harmful if swallowed (Acute Tox cat. 4), is suspected to cause cancer (cat. 2) and to be a mutagen (cat. 2), is a skin sensitiser (cat. 1), causes serious eye damage (cat. 1) and is very toxic to aquatic life (cat. 1).

The information available in the ECHA dossier includes more than 50 studies across different toxicity endpoints. Key studies and supporting evidence with a score of reliability of 1 or 2 were considered (a total of 40 studies, plus several in vivo toxicokinetic studies) (Online Resource 4).

Electrophilicity property partly explains the strong skin sensitising and mutagenic effect of hydroquinone (Madia et al. 2020). A number of studies, mainly new in vitro methodologies, available in the ECHA dossier for skin sensitisation and genotoxicity endpoints provide a substantial portion of the substance mechanistic information that align to almost all the KCs of carcinogens and, as such, inform other toxicity endpoints. We also reported toxicity information from the more recent Toxtracker in vitro genotoxicity assay that included a number of non-genotoxic endpoints (i.e., oxidative stress, protein damage, cellular stress/ER stress pathway) associated with increased cancer hazard thus, covering multiple KCs of carcinogens. Toxicity information provided by several in vivo studies especially for acute and repeated dose toxicity was not detailed. Neither ED properties nor immunotoxicity conventional studies were performed. The 2-year cancer study (OECD 453 2018) included in the dossier was instead, informative, providing also data on relevant biomarkers of exposure effect (DNA adducts, 8-OHdG bio-product), cell proliferation and morphology, apoptosis and other observations linked to the KCs. As for the two chemicals reported above, mechanistic information on tumour promotion, cell proliferation, DNA synthesis and lipid peroxidation specific to various target organs (e.g., urinary bladder, kidney, liver) were only provided by several additional non-standard studies included in the dossier. Those included also human studies, in vitro and epidemiological investigations (Online Resource 4).

Even if details of each single study were not available in the registration dossiers or in the assessment reports, it is evident that for a number of studies, mainly in vivo*,* there is a redundancy of similar observations and consistent lack of information in terms of observations for various endpoints. More importantly, the majority of mechanistic information is provided by additional non-standard studies not performed in compliance with GLP. Nevertheless, the information collected for the above substances showed that the observed effects provided by various studies across different toxicity endpoints can be indeed organised and integrated in a structured way on the basis of specific toxicity properties such as the ten KCs of carcinogens.

## New paradigms for sustainable safety testing

Despite being qualitative only, the approach presented here represents a 3D (three dimensional) reading of toxicity information that allows hazard to be evaluated by combining information from different systemic toxicity endpoints, rather than considering them individually.

The relevance of single observations provided across different studies and the degree of integration need to be based on mechanistic knowledge and biological plausibility. In this exercise, for example, the KCs of carcinogens have formed the mechanistic basis related to carcinogenicity and represented a pragmatic way to organise the information. This is also at the basis of the IATA for non-genotoxic carcinogens, recently developed by an OECD expert working group (Jacobs et al. 2016, 2020). There, overarching mechanisms and modes of action, identified from various cancer models, have been structurally organised with respect to the common hallmarks of cancer (Hanahan and Weinberg 2011) and the KCs of carcinogens (Smith et al. 2016).

The use of KCs to structure the information can be applied to any toxicity endpoint. In this regard, a number of KCs are now available for male and female reprotoxicants and endocrine disrupting chemicals (Arzuaga et al. 2019; Luderer et al. 2019; La Merrill et al. 2020). Interestingly, several of these characteristics are shared with those described for carcinogens (Smith et al. 2016). KCs of cardiotoxicants, neurotoxicants (NRC 2017) and immunotoxicants are under development as part of a collaborative project lead by University of California, Berkeley (https://keycharacteristics.org/). Furthermore, a JRC study in collaboration with University of Amsterdam is mapping the underlying mechanisms related to repeated dose toxicity to describe key characteristics of chemicals which are shown to induce systemic target organ effects. It is expected that for the above toxicities, provided that target organ specific toxicity mechanisms are included, a number of KCs will be also shared. When considering all together, there would be the possibility to highlight commonalities in terms of KCs of different systemic toxicants (e.g., to induce oxidative stress, to be genotoxic, be immunotoxic; induce chronic inflammation, modulate receptor-mediated effects) and specificities (e.g., immortalisation, cell transformation for carcinogenicity or altered spermatogenesis for male reprotoxicants) as well as in terms of test methods or sources of information to be used for evaluating them.

Given that the information is structurally (mechanism-based) organised as in our matrix, a number of questions can be addressed:

- Is a particular test necessary or is it redundant, in terms of provided information; is its potential fully exploited?
- Does the information provided by each test satisfy one or more of the key characteristics?
- What type of mechanistic study or source of mechanistic information can be integrated eventually to fill knowledge gaps?
- Is there opportunity to identify ways to enhance standard in vivo studies, on the basis of specific mechanisms, to maximise the information they provide?

These questions are particularly relevant from a regulatory perspective, especially in view of the new call for an enhanced risk assessment for all consumer products described in the EU Chemicals Strategy for Sustainability (European Commission 2020). To meet the possible increased requirements for safety assessment of such a large amount of new substances it would be necessary to optimise the collection, reporting and interpretation of toxicity information. Thus efficient (e.g., in terms of relevance and number of assays and, number of animals to be used) testing strategies may be also designed and applied to fulfil information requirements. This means focussing on an ad hoc selection of studies based on mechanistic understanding of biology and of specific toxicities thus avoiding overlaps and testing redundancies, as observed in the toxicological dossiers for the three examples illustrated here. In this respect, the matrix can serve as an example of how relevant information can be read across different test methods and toxicity endpoints. The identification of specific mechanisms and modes of action can help the selection of studies to be performed. The latter is also at the basis of the ongoing project promoted by the European Partnership for Alternative Approaches to Animal Testing (EPAA) that aims to develop a mechanism-based approach to cancer risk assessment for agrochemicals that uses targeted tools and test methods (Luijten et al. 2020; Heusinkveld et al. 2020). This approach could even go one step further if the classification of chemicals is based on a generic level of concern, rather than specific endpoints, as illustrated by Da Silva et al. (2020).

**Fig. 4:**
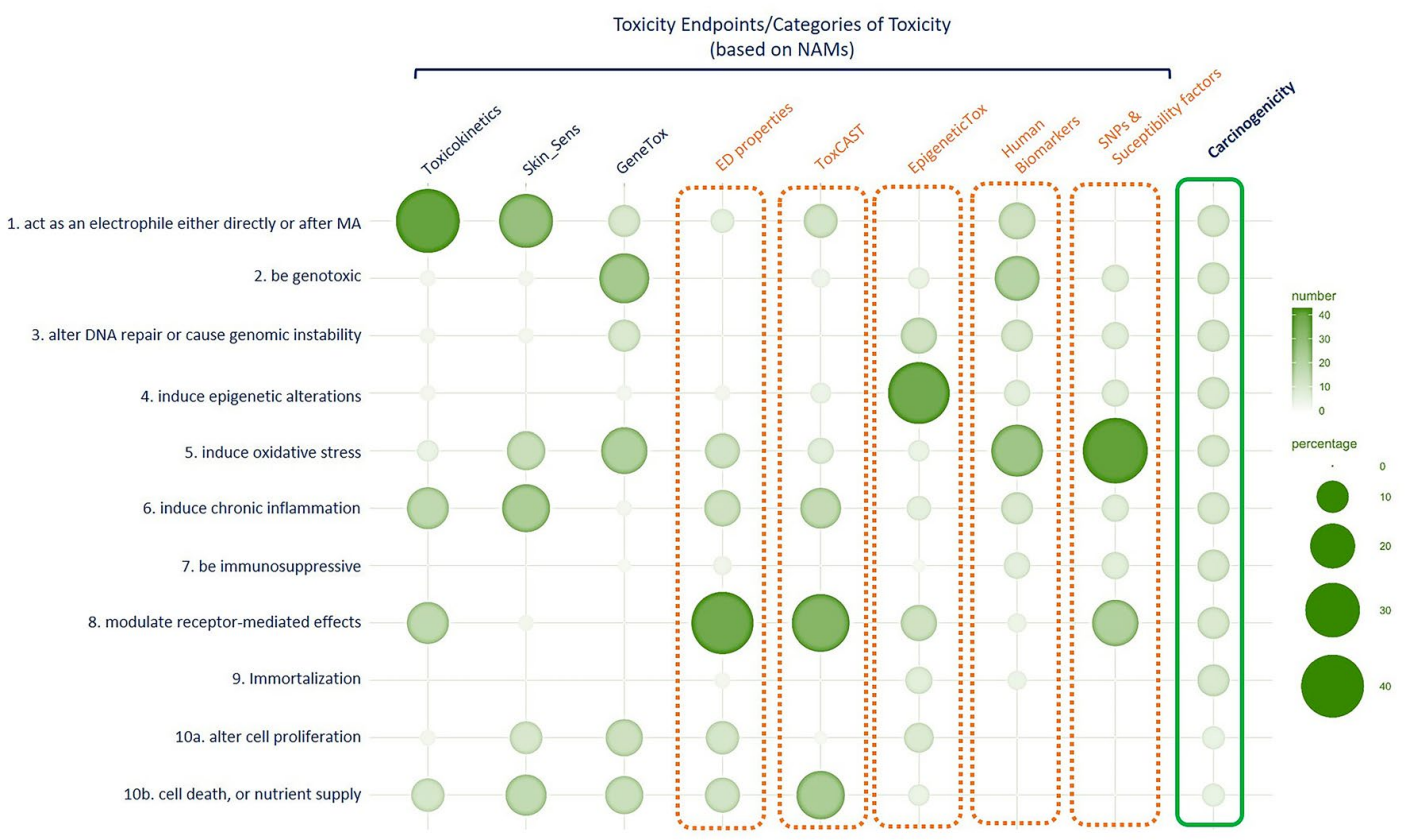
Hypothesis for a new paradigm for hazard assessment. Percentage distribution of toxicity information, provided by new approach methodologies (NAMs), potentially contributing to the ten key characteristics of carcinogens. The matrix is built on the observed effects provided by in silico, in vitro methods, AOPs, and recent models available for toxicokinetics, skin sensitisation, genotoxicity, more recent test protocols and/or investigative studies (orange dotted line) describing ED properties, epigenetic alterations (EpigeneticTox) or toxicity effects detected with HTS methodology (ToxCast data). Human relevant information on biomarkers of disease, SNPs (single-nucleotide polymorphisms) and susceptibility risk factors for disease was also included. Percent ratio for ToxCast data was calculated on the basis of selected assays as reported by Chiu et al. 2018

In relation to the example of carcinogenicity, we hypothesise that in the future toxicity prediction may be built solely on selected NAMs and integrated with human disease (cancer) related mechanistic information. The identification and use of specific molecular changes (fingerprints) has become an essential component for the characterisation of tumour development and progression. Fingerprints including biomarkers related to DNA, epigenetic, proteins and adducts can describe more directly mechanisms of carcinogenicity initiated by the exposure to environmental chemicals, thereby strengthening biological plausibility. They can also be applied to traditional epidemiological studies and be used as trackers to identify specific type of cancers (Ceccaroli et al. 2015; Grashow et al. 2018; Madia et al. 2019). In our hypothesis, we consider that three main toxicity endpoints such as toxicokinetics, skin sensitisation and genotoxicity may be supplemented with the inclusion of information for ED mode of action, ToxCast, EpigeneticTox, and human information for biomarkers and SNPs and susceptibility factors for cancer disease (Fig. 4). The selected information has been documented in regulatory guidance and OECD reviews (SCHER/SCCP/SCENIH 2009; OECD DRP 178 2012; Committee Carcinogenicity UK 2018; ECHA EFSA Guidance 2018). As shown in Fig. 4, this may indeed result in enrichment and a better distribution of the toxicity information based on mechanisms and specific biomarkers across the KCs of carcinogens. Such an approach may be valid following the understanding of exposure and considerations about dose–response relationships to correctly interpret the data, especially those provided by NAMs.

The NGRA based on NAMs, as recently shown by Baltazar et al. and Hatherell et al., exemplified this approach in a real scenario for systemic toxicity prediction in the context of the cosmetic industry (Baltazar et al. 2020; Hatherell et al. 2020). Interestingly, a number of biomarkers identified as essential in the cell stress panel designed within the NGRA, overlap with several biomarkers reported in our matrix which was based on the KCs of carcinogens (Table 1), highlighting further the possibility of cross-endpoint evaluation.

Even though the NGRA may be more readily accepted in the cosmetics sector, where the use of NAMs is the only testing option (Regulation (EC) 1223/2009), we consider that similar approaches can be designed and applied in a more holistic way in any testing setting across all systemic toxicity endpoints and across chemical sectors. However, for this purpose, it is considered that data integration, use of diverse sources of information and, the implementation of NAMs to be essential components.

## Supplementary Information

Below is the link to the electronic supplementary material.

Matrix of information across toxicity endpoints. The master table maps the available information, named as observed effects, provided from tests methods across toxicity endpoints to the ten key characteristics of carcinogens. The observed effects are categorised as reported in manuscript paragraph 2.3: red= indicated in the test protocol or in the test guideline as a specific endpoint result; yellow= embedded within the test protocol or the test guideline documents as a part of the information needed to obtain specific results or it could be extrapolated indirectly; dark green= derived from recent test protocols and/or investigative studies. The table includes also information on the product sector where specific test methods are required and the number of animals foreseen for each in vivo test method. Peer review papers and relevant documents consulted to build the master table are provided. Links to notes are embedded in the master table worksheet (XLSX 195 KB)

Matrix of information across toxicity endpoints for Linuron. The observed effects reported in the studies for Linuron (in blue and dark orange) have been overlaid to those of the master table reported in ESM_1. The observed effects are categorised as follow: blue = indicated in the test protocol or in the test guideline as a specific endpoint result; dark orange= embedded within the test protocol or the test guideline documents as a part of the information needed to obtain specific results or it could be extrapolated indirectly. The red colour of the master table has been changed to grey. The table includes also information on the product sector where specific test methods are required and the number of animals foreseen for each in vivo test method. Peer review papers and relevant documents consulted to build the master table are provided. Links to notes are embedded in the table worksheet. Chemical info card and overall analysis of the observed effects for Linuron reported in various studies across different toxicity endpoints are also reported (XLSX 788 KB)

Matrix of information across toxicity endpoints for 1,2-dichloroethane. The observed effects reported in the studies for 1,2-dichloroethane (in blue and dark orange) have been overlaid to those of the master table reported in ESM_1. The observed effects are categorised as follow: blue = indicated in the test protocol or in the test guideline as a specific endpoint result; dark orange= embedded within the test protocol or the test guideline documents as a part of the information needed to obtain specific results or it could be extrapolated indirectly. The red colour of the master table has been changed to grey. The table includes also information on the product sector where specific test methods are required and the number of animals foreseen for each in vivo test method. Peer review papers and relevant documents consulted to build the master table are provided. Links to notes are embedded in the table worksheet. Chemical info card and overall analysis of the observed effects for 1,2-dichloroethane reported in various studies across different toxicity endpoints are also reported (XLSX 565 KB)

Matrix of information across toxicity endpoints for hydroquinone. The observed effects reported in the studies for hydroquinone (in blue and dark orange) have been overlaid to those of the master table reported in ESM_1. The observed effects are categorised as follow: blue = indicated in the test protocol or in the test guideline as a specific endpoint result; dark orange= embedded within the test protocol or the test guideline documents as a part of the information needed to obtain specific results or it could be extrapolated indirectly. The red colour of the master table has been changed to grey. The table includes also information on the product sector where specific test methods are required and the number of animals foreseen for each in vivo test method. Peer review papers and relevant documents consulted to build the master table are provided. Links to notes are embedded in the table worksheet. Chemical info card and overall analysis of the observed effects for hydroquinone reported in various studies across different toxicity endpoints are also reported (XLSX 736 KB)

## Data Availability

Data and material are publically available.

## Abbreviations

DDI: Drug–drug interaction
EPA: US Environmental Protection Agency
EPAA: European Partnership for Alternative Approaches to Animal Testing
DAR: EFSA Draft Assessment Report
FDA: US Food and Drug Administration
CLP: Classification, Labelling and Packaging
GD: Guidance document
HTTr: High-throughput transcriptomics
IARC: International Agency for Research on Cancer
ICCR: International Cooperation on Cosmetics Regulation
KC: Key characteristic
MN: Micronucleus
NAMs: New approach methodologies
NGRA: Next generation risk assessment
OECD: Organisation for Economic Co-operation and Development
PBK: Physiologically based kinetic
RAR: EFSA Renewal Assessment Report
REACH: Registration, Evaluation, Authorisation and Restriction of Chemicals
TG: Test guideline
TK: Toxicokinetics

## Footnotes

1 Reliability of information. Klimisch codes. Score 1 = reliable without restrictions: “studies or data […] generated according to generally valid and/or internationally accepted testing guidelines (preferably performed according to GLP) or in which the test parameters documented are based on a specific (national) testing guideline […] or in which all parameters described are closely related/comparable to a guideline method.” Score 2 = reliable with restrictions: “studies or data […] (mostly not performed according to GLP), in which the test parameters documented do not totally comply with the specific testing guideline, but are sufficient to accept the data or in which investigations are described which cannot be subsumed under a testing guideline but, which are nevertheless well documented and scientifically acceptable”. Score 3 = not reliable: “studies or data […] in which there were interferences between the measuring system and the test substance or in which organisms/test systems were used which are not relevant in relation to the exposure (e.g. unphysiological pathways of application) or which were carried out or generated according to a method which is not acceptable, the documentation of which is not sufficient for assessment and which is not convincing for an expert judgment.” Score 4 = not assignable: “studies or data […] which do not give sufficient experimental details and which are only listed in short abstracts or secondary literature (books, reviews, etc.).” (ECHA R4, 2011).
